# Suitability of Real-Time PCR Methods for New Genomic Technique Detection in the Context of the European Regulations: A Case Study in Arabidopsis

**DOI:** 10.3390/ijms26073308

**Published:** 2025-04-02

**Authors:** Caroline Bedin Zanatta, Frank Narendja, Hilana El Jawhary, Gretta Abou-Sleymane, Saminathan Subburaj, Rubens Onofre Nodari, Sarah Zanon Agapito-Tenfen

**Affiliations:** 1 Department of Crop Science, Federal University of Santa Catarina, Rod. Admar Gonzaga 1236, Florianopolis 88034000, Brazil; carolinebedinzanatta@gmail.com (C.B.Z.); rubens.nodari@ufsc.br (R.O.N.); 2 Environment Agency Austria, Spittelauer Lände 5, 1090 Vienna, Austria; frank.narendja@umweltbundesamt.at; 3 Faculty of Health Sciences, American University of Science and Technology, Ashrafieh, Alfred Naccache Avenue, Beirut P.O. Box 16-6452, Lebanon; hjawhary@aust.edu.lb (H.E.J.); gabousleymane@aust.edu.lb (G.A.-S.); 4 NORCE Norwegian Research Centre AS, Climate & Environment Department, Siva Innovasjonssenter, Postboks 6434, 9294 Tromsø, Norway; sasu@norceresearch.no

**Keywords:** genetically modified organism, gene editing, CRISPR, single nucleotide polymorphism, single point mutation, new genomic techniques

## Abstract

PCR methods are widely applied for the detection of genetically modified organisms (GMOs) in Europe, facilitating compliance with stringent regulatory requirements and enabling the accurate identification and quantification of genetically modified traits in various crops and foodstuffs. This manuscript investigates the suitability of real-time PCR methods for detecting organisms generated through new genomic techniques (NGTs), specifically focusing on a case study using *Arabidopsis thaliana* as a model gene-edited plant. Given the complexities of European regulations regarding genetically modified organisms (GMOs) and the classification of gene-edited plants, there is a pressing need for robust detection methods. Our study highlights the development and validation of a novel single-plex real-time PCR method targeting a specific single nucleotide polymorphism (SNP) in the *grf1-3* gene modified using CRISPR-Cas9 technology. We emphasize the effectiveness of locked nucleic acid (LNA)-modified primers in improving specificity. The results demonstrate that while the *grf1-3* LNA method successfully detected and quantified gene-edited Arabidopsis DNA, achieving absolute specificity remains a challenge. This study also addresses the significance of the cross-laboratory method for validation, demonstrating that the method developed for an SNP-modified allele can be performed in accordance with the precision and trueness criteria established by the European Network of GMO Laboratories (ENGL). Furthermore, we call for continued collaboration among regulatory agencies, academia, and industry stakeholders to refine detection strategies. This proactive approach is essential not only for regulatory compliance but also for maintaining public trust in the safe integration of gene-edited organisms into food products.

## 1. Introduction

PCR detection methods have become essential tools for the identification of GMOs. With the advent of techniques like CRISPR-Cas9, scientists can introduce single point mutations with remarkable precision. A new generation of GM plants is reaching the market with trait phenotypes provided by point mutations introduced through NGTs, such as gene editing.

In the European Union, the regulation of GMOs is primarily governed by Directive 2001/18/EC and Regulation (EC) No. 1829/2003, which mandate comprehensive risk assessments and labeling for organisms and food resulting from genetic modification [1]. However, the application of these regulations to gene-edited plants is complex and often debated. The Court of Justice of the European Union (CJEU) ruled in 2018 that organisms obtained by mutagenesis, including gene editing techniques, should be classified as GMOs unless they result in changes that could have occurred naturally. This legal framework necessitates robust detection methods to identify and quantify the GMO in place [2].

PCR detection methods are particularly well-suited to identify specific single point mutations in gene-edited organisms and their derivative foodstuffs. These methods can be designed to amplify target DNA sequences with precision, enabling the differentiation between edited and wild-type alleles. Techniques such as real-time quantitative PCR (qPCR) and high-resolution melting analysis (HRM) allow for the quantification of the gene-edited variants and the detection of minor variations. By designing primers that flank the edited region, researchers can effectively identify whether a particular mutation exists, facilitating compliance with EU regulations concerning the traceability and labeling of GMOs [3,4,5].

Despite the advantages of PCR techniques, challenges remain in the detection of gene-edited plants within the context of European law. One major issue is the variability in mutation loci and the potential presence of such mutations in other species, varieties, and organisms, which can complicate the validation of assays for routine food market and environmental monitoring. Furthermore, the development of detection methods must keep pace with the rapid evolution of gene editing technologies, as new techniques may introduce mutations in ways that existing validated assays cannot detect. The need for continuous improvement and standardization of detection methods is crucial for regulatory compliance and consumer confidence [6].

Recent experimental studies have introduced promising detection methods for gene-edited plants, highlighting that droplet digital PCR (ddPCR) combined with standard PCR primer/probe systems, along with targeted next-generation sequencing (NGS), provide superior performance compared to conventional real-time PCR (qPCR) [7,8,9,10,11,12]. As of now, a specific qPCR detection method has only been developed for one commercialized gene-edited product: the herbicide-tolerant oilseed rape (OSR) created by the US company Cibus [13]. Nonetheless, there remains substantial debate regarding the efficacy of this method, particularly concerning its event-specificity and its suitability for monitoring the presence of this EU-unauthorized NGT product in the European market [14].

In this study, the development and validation of a novel single-plex real-time PCR method that targets a single point mutation in Arabidopsis is presented as a case study for the discussion on the suitability of qPCR methods for NGT plants in general. Our method falls within the gray area of amplification of wild-type genotypes, or genotypes containing the wild-type sequence, at high cycle threshold (Ct) levels, leading to different interpretations of positive or negative results. This study shows that real-time PCR can be suited for NGT plants, but on a case-by-case analysis, depending on the genomic context of the introduced genetic change, the host species, and the availability of genomic databases allowing in silico investigation of target sequence similarities.

## 2. Results

### 2.1. Quantitative PCR Method Using Unmodified Taqman™ Primers and Probe

**Figure 1:**
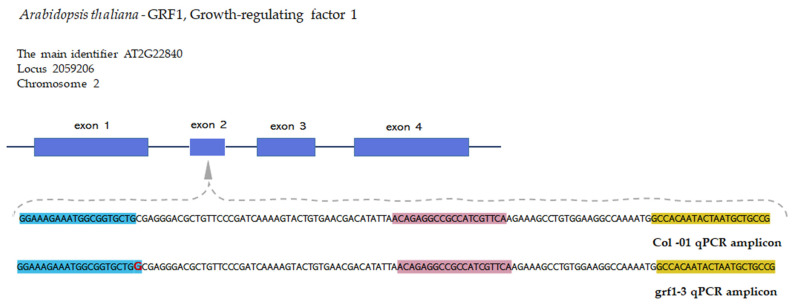
GRF1 gene structure representing the four exons obtained from the *Arabidopsis thaliana* Columbia-0 genotype. Blue boxes indicate the exons in the amplicon sequence for Columbia-0 ecotype (unmodified parental line) and oligonucleotide primer set shown in different colors. The position of the single nucleotide polymorphism in the genomic locus of *grf1-3* the Arabidopsis NGT genotype (in red bold). Blue highlighted sequences are oligonucleotide forward sequences, pink are probe sequences and yellow are reverse sequences.

The unmodified oligonucleotide primer set was first tested in several assays to determine the ideal concentration of DNA, cycling parameters, denaturation, and annealing temperatures. Figure 1 shows the location of the oligonucleotide primer set in exon 3 of the *grf1-3* gene.

**Figure 2:**
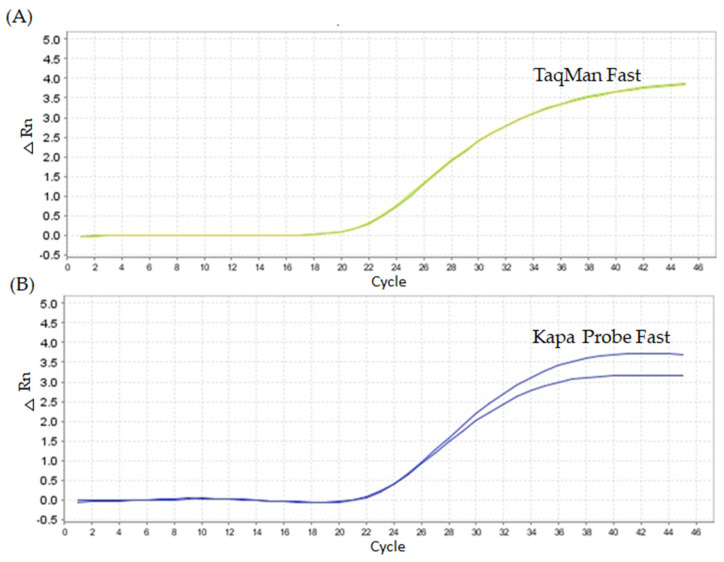
PCR amplification plot of *Arabidopsis thaliana* *grf1-3* using two different master mixes and the *grf1-3* unmodified primer set. Figure (**A**) TaqMan Fast Advanced Master Mix and (**B**) Kapa Probe Fast qPCR Master Mix (2×). The conditions of the oligonucleotide’s primer were set the same. The cycling program used was 95 °C for 10 min, 95 °C for 2 s, 60 °C for 20 s.

We employed two different master mixes: (i) TaqMan™ Fast Advanced Master Mix and (ii) Kapa Probe Fast qPCR Master Mix (2×), which yielded contrasting results. The first master mix exhibited a higher Cq value (22.67) in *grf1-3* DNA compared to the second master mix tested under the same conditions (Cq 20.90). Both master mixes produced the same fluorescence (ΔRn 3.5), but a lower Cq value was obtained with Kapa Probe Fast, which was subsequently used for the following experiments (Figure 2).

**Figure 3:**
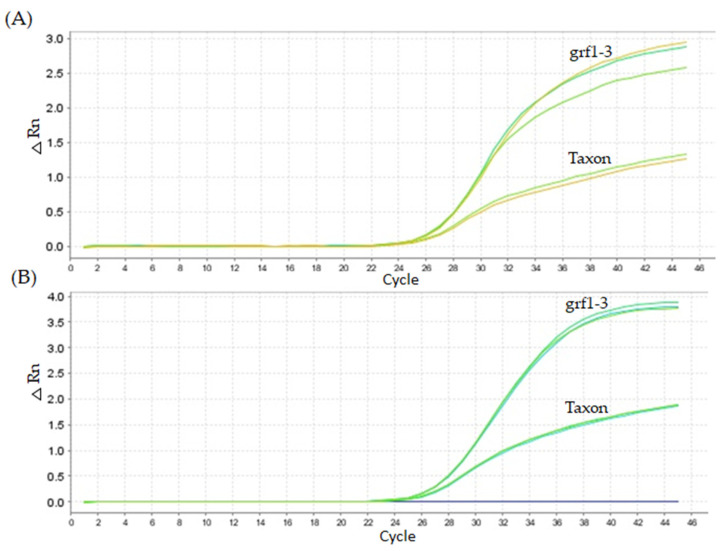
Performance of grf-1 and taxon-specific PCR under different cycling conditions. The concentration of the oligonucleotide primers were set the same (200 nM forward and 200 nM reverse, probe 200 nM) in the PCR reaction (2.94 ng). Figure (**A**) Kapa’s manufacturer cycling recommendations (Program 1: 95 °C for 10 min, 95 °C for 2 s, 60°C for 20 s) and (**B**) thermocycling method commonly used for GMO target detection (Program 2: 95 °C for 10 min, 95 °C for 15 s, 60 °C for 1 min).

Two qPCR cycling conditions were assessed, one based on Kapa’s manufacturer recommendations (Program 1: 95 °C for 10 min, 95 °C for 2 s, 60 °C for 20 s) and a second condition for a general program (Program 2: 95 °C for 10 min, 95 °C for 15 s, 60 °C for 1 min) (Figure 3). The Cq values were very similar between the two cycling methods. The primary difference between the cycling conditions was observed in fluorescence intensity. Using the Kapa master mix cycling method, the ΔRn for the taxon gene was 1.0 and for the *grf1-3* oligo set was 2.5. In contrast, with the ENGL method, the fluorescence signal was higher (taxon gene, ΔRn of 1.5; *grf1-3* oligo set, ΔRn of 3.5).

Assays for DNA concentration were based on nominal copy numbers (10,000 and 20,000 copies) considering the *Arabidopsis thaliana* genome size. Two titration tests were conducted for both copy number samples.

**Figure 4:**
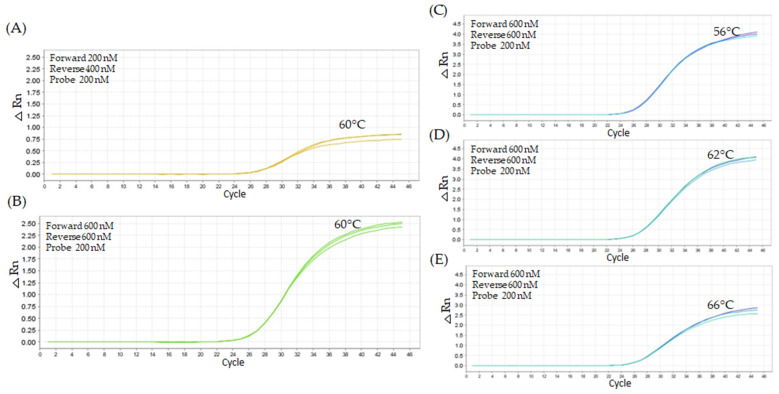
Performance of *grf1-3* and taxon-specific PCR amplification under different concentrations for the unmodified TaqMan *grf1-3* primer set. PCR amplification plots for *Arabidopsis thaliana* *grf1-3* genotype DNA (10,000 copies). Figure (**A**) shows the less favorable primer combination, while (**B**) shows the most favorable combination at 60 °C, using a constant probe concentration of 200 nM. Figures (**C**–**E**) display the amplification profiles for the *grf1-3* primer set at optimal concentrations (600 nM forward primer, 600 nM reverse primer, and 200 nM probe) across a range of annealing temperatures (56 °C to 66 °C). Amplification was performed using Kapa Probe Fast qPCR Master Mix (2×) under cycling conditions of 95 °C for 10 min, followed by 45 cycles of 95 °C for 15 s and annealing at a variable temperature (°C) for 1 min.

The amplification of 10,000 copies of *Arabidopsis thaliana* was conducted using the unmodified TaqMan *grf1-3* primer set employing different concentrations of forward and reverse primers (200 nM, 400 nM, and 600 nM). At 60 °C, the amplification profiles with 200 nM forward primer and 400 nM reverse primer resulted in a less satisfactory curve, with an average Cq value of 26.74 (Figure 4A). An improvement in amplification efficiency was detected with increased primer concentrations, particularly at 600 nM for both primers (Figure 4B). The titration of the probe (100 nM, 200 nM, and 300 nM), with forward and reverse primers at 600 nM, indicated that the probe concentration of 200 nM, at an annealing temperature of 62 °C (Figure 4D), exhibited the optimal balance between amplification efficiency and curve fitting, resulting in a Cq value of 25.82 and a fluorescence ΔRn of 2.5.

**Figure 5:**
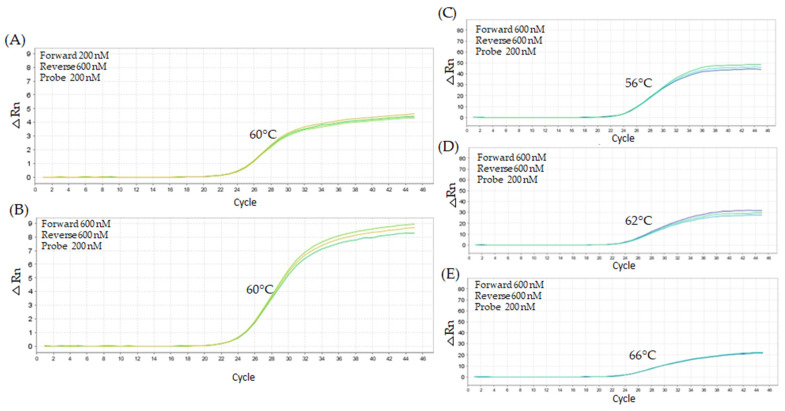
Performance of *grf1-3* and taxon-specific PCR amplification under different concentrations for the unmodified TaqMan *grf1-3* primer set. PCR amplification plots for *Arabidopsis thaliana* *grf1-3* genotype DNA (20,000 copies). Figure (**A**) shows the less favorable primer combination, while (**B**) shows the most favorable combination at 60 °C, using a constant probe concentration of 200 nM. Figures (**C**–**E**) display the amplification profiles for the *grf1-3* primer set at optimal concentrations (600 nM forward primer, 600 nM reverse primer, and 200 nM probe) across a range of annealing temperatures (56 °C to 66 °C).

A second titration test was conducted using 20,000 copies of *grf1-3* DNA and varying concentrations of oligonucleotide primers (200 nM, 400 nM, and 600 nM) to confirm the consistency of the previous results (Figure 4). The combination of a 200 nM forward primer and 600 nM reverse primer produced the highest Cq value of 20.57 (Figure 5A), indicating less favorable amplification. The best amplification, with the most optimal curve shape, was achieved using 600 nM of both forward and reverse primers, yielding a Cq value of 20.77. The forward and reverse probe at 600 nM across probes concentrations (100 nM, 200 nM, 300 nM) and temperatures ranging from 56 °C to 66 °C showed minimal variation in amplification efficiency. At 56 °C, the 200 nM probe concentration resulted in an average Cq value of 23.19, while at 62 °C, the same probe concentration yielded a slightly lower Cq value of 23.59. At 62 °C, using a 300 nM probe concentration, the Cq value was 23.33, similar to the result obtained with 200 nM. Overall, consistent amplification and fluorescence responses were observed across all tested temperatures (56 °C to 66 °C), with each temperature showing a distinct pattern and minimal influence from probe concentration (Figure 5C–E).

**Figure 6:**
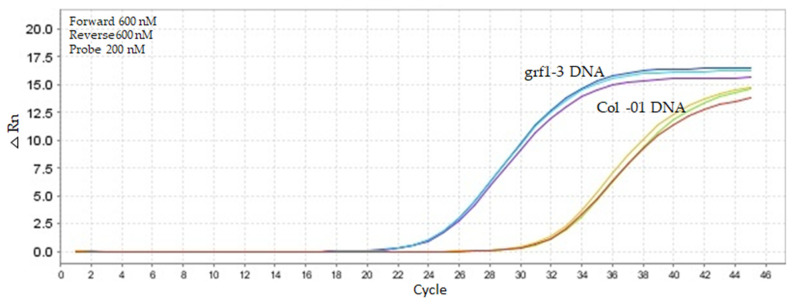
The performance specificity test of *grf1-3* and Col-01 templates using 20,000 copies with the unmodified TaqMan *grf1-3* primer set. Identical primer and probe concentrations were used for both DNAs (600 nM forward, 600 nM reverse, and 200 nM probe). Amplification was performed using Kapa Probe Fast qPCR Master Mix (2×) under cycling conditions of 95 °C for 10 min, followed by 45 cycles of 95 °C for 15 s and annealing at 60 °C for 1 min.

Under adjusted conditions (600 nM forward and 600 nM reverse primer, 200 nM) using 20,000 copies of template DNA at 62 °C. The standard curve assay with a range from 20,000 copies (Cq 23.34) to 2 copies (Cq 37.01) (Supplementary Material S1) produced an amplification efficiency of around 95.4%, with an R^2^ value of 0.9995 and a slope of −3.43. Primer specificity assessed empirically using TaqMan set *grf1-3* primers resulted in amplification with a Cq of 21.44 and a ΔRn of 17.5 in the *grf1-3* template (20,000 copies). In comparison, amplification with the Col-01 template (20,000 copies) using *grf1-3* target primers yielded a Cq of 29.6 and a ΔRn of 15 (Figure 6). The consistent amplification for both DNA templates indicates that the TaqMan primer set failed to discriminate the CRISPR/Cas9 line from Col-01, the control genotype.

### 2.2. Quantitative PCR Method Using Modified LNA Primers

#### 2.2.1. First Titration Assay—Lower Oligonucleotide Concentrations

**Figure 7:**
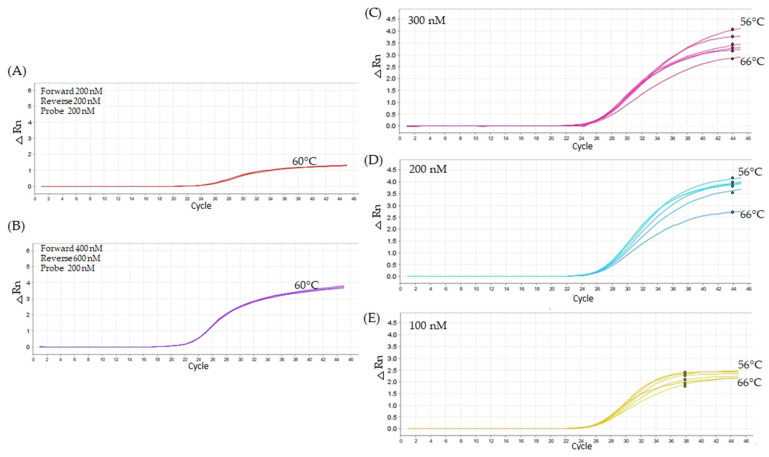
The performance of the primers under various experimental conditions. Panels a and b show the results from cycling conditions of 95 °C for 10 min, followed by 45 cycles of 95 °C for 15 s and annealing at 60 °C for 1 min. Panel (**A**) shows the less favorable result observed during the titration, while Panel (**B**) highlights the most favorable result. Panels (**C**–**E**) illustrate the performance of the primer sets at the optimal concentrations (400 nM forward and 600 nM reverse), tested with different probe concentrations (100 nM, 200 nM, and 300 nM). Furthermore, the assays were assessed at varying temperatures of 56 °C, 62 °C, and 66 °C, as illustrated in Figure 5C–E, respectively.

A lack of specificity for *grf1-3* demonstrated by cross-reactivity with Col-0 using the unmodified TaqMan method highlighted the need for improved specificity in SNP detection via modified qPCR. To address this, we modified the forward primer using locked nucleic acid (LNA) technology. The assay utilized 20,000 copies of Arabidopsis haploid DNA (2.94 ng in 5 µL) with varying concentrations of oligonucleotide primers (200 nM, 400 nM, and 600 nM) that produced approximate Cq values between 21 and 26 cycles (Table 1). A 200 nM forward primer and 200 nM reverse primer produced a less favorable result, with a Cq of 26.02 (Figure 7A). The most favorable results were achieved using 400 nM forward and 600 nM reverse primers, yielding a Cq value of 21.49 (Figure 7B). Forward and reverse (400 nM forward, 600 nM reverse, and 200 nM probe) were tested at probe concentrations (100 nM, 200 nM, 300 nM) and temperatures ranging from 56 °C to 66 °C. The 200 nM concentration was used between the temperatures, demonstrating better amplification efficiency at 62 °C. (Figure 7D). As a result of the optimized conditions (400 nM forward, 600 nM reverse, and 200 nM probe at 62 °C), the Col-01 DNA tested for its specificity to the LNA primer *grf1-3* produced Cq values around 35–37, while the *grf1-3* genotype DNA had a Cq value of 21.61. Based on the information that the concentration of the LNA primer changes the Cq value and the need for increased sensitivity for detection of SNP, an additional round of titration was performed.

#### 2.2.2. Second Titration Assay—Higher Oligonucleotide Concentrations

**Figure 8:**
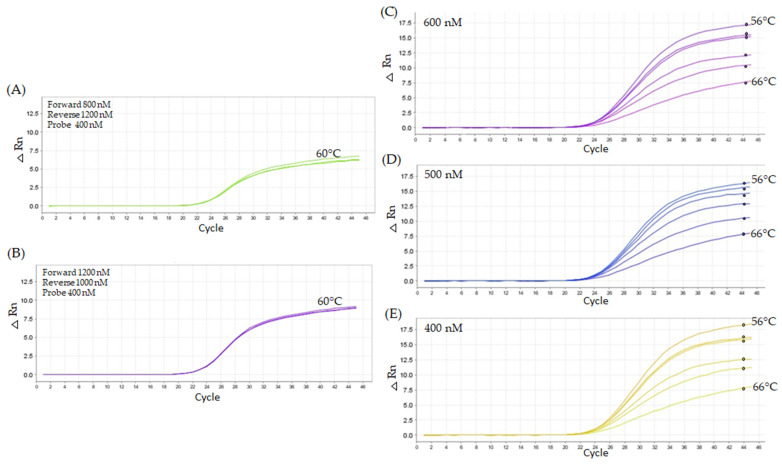
The figure illustrates the performance of the primers under various experimental conditions. Panel (**A**) displays the least favorable outcome observed during the titration, while panel (**B**) presents the most favorable result, both using a constant probe concentration of 400 nM. The results were obtained under cycling conditions of 95 °C for 10 min, followed by 45 cycles of 95 °C for 15 s and annealing at 60 °C for 1 min. Panels (**C**–**E**) highlight the performance of primer concentrations of 1200 nM forward and 1000 nM reverse primers, tested with varying probe concentrations of 400 nM, 500 nM, and 600 nM evaluated across a range of annealing temperatures, from 56 °C to 66 °C.

The subsequent titration used forward and reverse primers at higher concentrations (800 nM, 1000 nM, and 1200 nM) while maintaining the probe concentration at 400 nM. Overall, the primer combinations exhibited similar patterns in Cq values, although fluorescence intensity exhibited alteration across the increasing primer concentrations (Table 2). The combination considered least favorable for Cq, using 800 nM forward primers and 1200 nM reverse primers, resulted in a Cq value of 23.93, with peak fluorescence near ΔRn 5 (Figure 8A). In contrast, pairing 1200 nM forward primers with 1000 nM reverse primers produced the most favorable result, yielding an average Cq of 23.37 and peak fluorescence near ΔRn 10 (Figure 8B). When oligonucleotide concentrations (1000 nM forward and 1200 nM reverse) combined with probe concentrations (400 nM, 500 nM, and 600 nM) across temperature gradients ranging from 56 °C to 66 °C were used, intriguing results were found. For example, the intermediate concentration (500 nM) was most sensitive to the temperature gradient tested (Figure 8D). The 400 nM probe concentration demonstrated the best performance, with Cq values showing minimal variation across the tested concentrations and remaining more stable concerning temperature fluctuations. At 62 °C, with 1000 nM forward and 1200 nM reverse primers, the 400 nM probe concentration yielded the best results (Cq 23.62) compared to the 500 nM (Cq 23.78) and 600 nM (Cq 23.83) concentrations (Figure 8C–E).

#### 2.2.3. Third Assay—Replication of Best Titration Results

**Figure 9:**
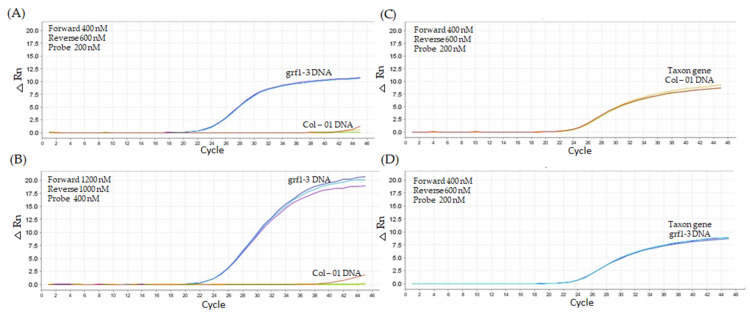
The LNA and taxon primer sets were conducted under the following cycling conditions: an initial denaturation at 95 °C for 10 min, followed by 45 cycles of 95 °C for 15 s, and annealing at 62 °C for 1 min. The analysis used a DNA template containing 20,000 copies (2.94 ng per PCR reaction). Panel (**A**) presents the amplification profiles for Col-01 and *grf1-3* DNA using the LNA primer set (400 nM forward, 600 nM reverse, and 200 nM probe). Panel (**B**) shows the amplification profiles for Col-01 and *grf1-3* edited DNA with the LNA primer set (1200 nM forward, 1000 nM reverse, and 400 nM probe). Panels (**C**,**D**) display the results for Col-01 and *grf1-3*, respectively, using the taxon primer set (400 nM forward, 600 nM reverse, and 200 nM probe).

To verify the consistency of results from the best-performance titration tests, we conducted a third experiment using a template containing 20,000 copies of unmodified Col-01 DNA and *grf1-3* edited DNA. In this experiment, we tested the *grf1-3* LNA system (600 nM forward primer, 600 nM reverse primer, and 200 nM probe) with Col-01 template DNA (Table 2), and we observed an average Cq value close to 40 cycles with a ΔRn of approximately 1 (Figure 9A). Amplification was detected for the *grf1-3* gene in the edited DNA, yielding a Cq value of 21.15 and a ΔRn of 10. Secondly, we tested 1000 nM forward primer, 1200 nM reverse primer, and 400 nM probe against the Col-01 DNA, which resulted in a Cq value of approximately 39 cycles, with two undetermined results out of three replicates (Figure 9B). For the *grf1-3* edited DNA, consistent amplification was observed with a Cq value of 21.14.

The key difference between the titrations was the higher fluorescence and lower Cq observed in the second test compared to the first. The standard curve, generated from triplicates at concentrations ranging from 20,000 copies to 0.2 copies per 5 µL sample, exhibited a slope of y = −3.437x + 40.425 and an R^2^ value of 0.9995, indicating assay consistency. The taxon primer system under standard conditions (400 nM forward primer, 600 nM reverse primer, and 200 nM probe) successfully amplified both Col-01 DNA (Figure 9C) and *grf1-3* DNA (Figure 9D), with Cq values of 22.45 and 22.03, respectively.

### 2.3. Interlaboratory Validation Results

#### 2.3.1. Limit of Detection (LOD)

The *grf1-3* method was tested with six dilution levels of the target *grf1-3* DNA (40, 20, 10, 3, 1, and 0.1 copies) while maintaining a constant concentration of non-target DNA (salmon sperm DNA at 20 ng/µL) (Table 3). Results from the laboratory revealed that the 95% limit of detection (LOD_95%) was established at five genomic copies, based on 12 PCR replicates at each dilution level. Consequently, the LOD_abs was confirmed to be less than 25 copies per PCR reaction with 95% confidence, ensuring that no more than 5% of the results were false negatives.

#### 2.3.2. Robustness

Robustness testing was conducted according to the guidelines provided by the German Federal Office of Consumer Protection and Food Safety (BVL) [15] as the ENGL guidance document [16], simulating real-world conditions to evaluate sensitivity under minor deviations from standard experimental protocols. The method was evaluated through testing on ABI 7500 and Bio-Rad CFX96 qPCR machines utilizing two different master mixes: KAPA PROBE FORCE qPCR Master Mix and QuantiTect Multiplex qPCR Kit. Modifications included alterations in PCR master mix volume (19 and 21 µL with 5 µL of sample DNA), annealing temperatures (±1 °C), master mix volumes (±5%), and reductions in primer and probe concentrations by 30% (Table 4).

Cq values for the *grf1-3* primer ranged from 35.24 to 36.09 across the eight experimental conditions. Comparable results were achieved with both master mixes, indicating robustness across different conditions. Positive results were obtained for all replicates under each variation, demonstrating compliance with the robustness criteria established by BVL [17].

Further sensitivity analyses, performed using probability of detection (POD) curve modeling, confirmed the method’s robustness [18]. A probability of detection (LOD_95%) of 1.664 was established, with a 95% confidence interval ranging from 0.887 to 3.108.

#### 2.3.3. Trueness and Precision

To ensure accuracy and precision, we evaluated both intra-assay (replicates within individual assays) and inter-assay (replicates across different runs) variability by analyzing DNA with genetically modified (GM) content of 0.1%, 1%, and 10% relative to a taxon primer set using the *grf1-3* primers. Trueness and precision were evaluated through repeatability tests involving four qPCR runs for four different DNA extracts of each GM level. Each PCR experiment was performed in 10 replicates at concentrations near the expected limit of detection (LOD), as detailed in reference [16]. The trueness and precision of the PCR method were assessed according to predetermined criteria for accuracy (±25%) and precision (RSDr ≤ 25%).

The EAA laboratory measured the *grf1-3* target concentration to be approximately 0.09%, slightly lower than the expected value of 0.1%, with an RSDr of 15.10%, thus meeting the minimum performance criteria established by ENGL (Table 5). For the target concentration of 1%, the GM portion was quantified as 1.106% with an RSDr of 7.58%, aligning with ENGL criteria. At the target GM level of 10%, the *grf1-3* concentration measured was 10.154% with an RSDr of 6.16%, consistently performed according to the established criteria across all tested concentrations, exhibiting accuracy at lower concentrations. These results indicate that the qPCR method can be effective for NGT detection and quantification, where the detection of SNPs underscores the challenges of maintaining consistency across testing environments when using qPCR.

#### 2.3.4. Limit of Quantification (LOQ)

The LOQ was assessed by generating a standard curve using genomic copies of target *grf1-3* DNA, ranging from 2500 to 40 copies, with a constant concentration of non-target DNA (salmon sperm DNA at 20 ng/µL).

The lowest concentration observed with acceptable trueness and precision levels was the 40 genomic copy level point, with RSDr values at 20.6% (Table 6). As the copy number in the PCR reaction decreased, RSDr values correspondingly increased, highlighting the challenges associated with maintaining precision at lower concentrations. For the lowest genomic copy levels tested (specifically, 1 and 0.1 copies), the RSDr values were 58.3% and 16.5%.

Surprisingly, the number of copies measured was 10 times higher than expected for the 0.1 copies level (1.2 copies). These results suggest problems in the standard solution formulation at very low-level samples.

### 2.4. In Vitro Specificity Testing for the Newly Established qPCR Method

Empirical testing was conducted to evaluate cross-reactivity with non-target DNA sequences in genetically modified (GM) and non-GM oilseed rape (*Brassica napus*) and *Arabidopsis thaliana* genotypes. The analysis using the *grf1-3* PCR system involved oilseed rape samples of accessions named GT73, MS8, RF3, and 88302, which exhibited non-specific amplification signals, as detailed in Table 7. Testing of *Arabidopsis thaliana* genotypes, named grf3-9, grf4-17, and grf8-61, showed similar results (Table 8).

## 3. Discussion

### 3.1. Suitability of Enhanced Real-Time PCR Systems for NGT Detection

The advent of site-specific nucleases, such as CRISPR/Cas9, has transformed genetic manipulation while also posing challenges for the identification and quantification of GMOs in food and in the environment. This study evaluated the effectiveness of real-time qPCR method targeting a CRISPR-generated mutation through LNA modifications in the reverse oligonucleotide primer sequence.

Previous in silico analyses showed potential cross-reaction as the *grf1-3* primer sequences exhibited substantial complementarity with Brassica species [6]. Although mismatches were detected in both the forward and reverse primers when tested against *Brassica napus*, there was a need to ensure primer specificity for this method. Therefore, LNA modification was introduced in this method, and an in vitro assay was developed in this study. The outcomes of a series of PCR assays show that the oligonucleotide primer set successfully amplified *Arabidopsis thaliana* *grf1-3* DNA. On the other hand, even through the application of the LNA strategy, the primer set exhibited limited differentiation from the wild type unmodified parental line Col-01 but also between genotypes of different genus, which is expected for highly conserved sequences for the GRF gene. The titration experiments revealed residual amplification at high Cq values when using Col-01 DNA.

The complexity involved in developing and validating qPCR methods for GMOs is evident from our findings and corroborated by the literature. Similarly to our results, Chhalliyil and collaborators [13] found that while LNA-modified primers could enhance specificity, they sometimes yielded amplification at high cycle thresholds (Cq > 41).

Our validation study established a limit of detection (LOD95%) of approx. five genomic copies, consistent with methods demonstrated by Weidner and colleagues [19], which reported an LOD95% of approximately five copies/PCR for AgroBorder I, three copies/PCR for AgroBorder II, and five copies/PCR for P-CsVMV-pat varieties in multiplex real-time PCR methods. Similarly, Fraiture et al. [7] detected positive signals ranging from 5 to 10 copies for a mutant rice-edited organism using digital PCR probe strategies.

The robustness of the *grf1-3* method was evaluated across different qPCR platforms (ABI 7500, ThermoFisher Scientific, Vienna, Austria; and Bio-Rad CFX96, Bio_rad Laboratories, Vienna, Austria) and master mixes(KAPA PROBE FORCE and QuantiTect Multiplex. Additionally, precision and trueness were assessed at three concentration levels (0.1%, 1%, and 10%) with RSDr levels below 25%, indicating that the method provides consistent and reproducible results with accuracy across different concentrations. Method validation showed a limit of quantification (LOQ) of 40 genomic copies with an average RSDr of 20.6% (relative standard deviation ≤ 25%) and trueness ±25% in both laboratories (POD around 1.6). These results are comparable to those obtained using gene-edited SU canola DNA, reaching the ENGL acceptance criteria [13,14,19].

Despite an overall good performance of the method developed in this study, the issue of specificity remains a major obstacle. Zhang and colleagues [20] showed that small DNA variations could lead to ambiguous amplification signals. In their study, primer/probe combinations produced typical amplification curves from wild-type rice DNA and weak signals from other edited DNA genotypes (CAO1-2, CAO1-3, CAO1-4, and CAO1-7). Similarly, Weidner and colleagues [14] found non-specific amplification signals of herbicide-resistant canola genetically modified by oligonucleotide-directed mutagenesis. PCR amplification using an LNA set of primers showed Cq values around 40 in wild-type DNA templates (AHAS1C-WT, AHAS3A-WT, and AHAS3A-SU&CL). Weidner et al. [19] found unexpected signals (Cq ≥ 35) for the AgroBorder I PCR method when applying it to the GM cotton event MON88701 DNA.

These findings underscore the complexity of achieving absolute specificity, particularly for single nucleotide modifications when the technique used for detection and quantification is based on PCR amplification strategies. In this sense, PCR methodologies can still serve the purpose for some NGT events depending on the nature and complexity of the modified gene(s).

The qPCR sensitivity, additionally, is a critical factor. Our titration results reveal that with 20,000 DNA copies, differences in Cq values across oligonucleotide primer concentrations remain minimal. At lower DNA copy numbers, primer concentration significantly influences sensitivity. In the second titration assay, the intermediate primer concentration (1000 nM) showed Cq sensitivity to temperature variations. The effect could be because of the susceptibility of differential binding energies of the oligonucleotides, particularly at higher annealing temperatures and GC-rich templates, such as those derived from plant genomes [21].

However, there is a constant need for method refinement and technical improvement to ensure accurate differentiation between similar sequences with distinct agricultural traits [22]. The difficulties encountered include the variability in Cq values and fluorescence signals, especially at lower GM concentrations. Contributing factors may include the inherent complexity of the DNA matrix, potential primer–dimer formations, and insufficient machine capability and laboratory routines.

### 3.2. Advantages of Other Analytical Methods for NGT Detection

Gene-edited organisms have not yet been approved for commercial use within the European market; however, the adoption of new genomic techniques (NGTs) for plant breeding programs is progressing worldwide, including in the Americas, Africa, Asia, and Oceania [8]. This situation highlights the urgent need to advance and enhance detection techniques for these novel organisms.

Recent advancements in PCR-based assays, such as loop-mediated isothermal amplification [23], ligase chain reaction [24], the incorporation of RNase H2 in rhAmp technology [25,26], and high-resolution melting [27], demonstrate improvements in detection capabilities.

In light of the continuous evolution in biotechnology, Digital Droplet PCR (ddPCR) has gained recognition for its ability to detect and quantify low-abundance DNA in NGTs. This approach enables the detection of a single DNA copy without the necessity for reference materials or standard curves, providing high precision [7]. The development of a duplex ddPCR method for detecting gene-edited rice lines has demonstrated effectiveness in achieving high specificity for two distinct events [7,20].

To address the detection challenges, evolving detection strategies is essential. Screening for the CRISPR/Cas machinery, particularly *Streptococcus pyogenes* Cas9 (SpCas9), represents a targeted approach for identifying organisms harboring integrated CRISPR/Cas9 vectors, especially for detecting stably integrated gene-editing constructs [28,29,30,31]. High-Throughput Sequencing (HTS) offers a comprehensive view of genetic alterations, including complex rearrangements, point deletions, and the integration of exogenous DNA. This method has proven effective in detecting NGT-derived products in crops such as rice and rapeseed [32,33]. Sequencing can enhance detection sensitivity and specificity, as demonstrated in rice studies using Illumina technology [8]. Despite the high costs and lengthy processing times associated with WGS, its comprehensive approach is invaluable for identifying and characterizing gene-edited organisms. Techniques such as SMRT-OTS and Nano-OTS can provide greater detail on CRISPR/Cas9 modifications in genomes [34].

PacBio HiFi sequencing, which operates based on DNA polymerase activity, offers a promising alternative. In this technique, DNA polymerase binds to template DNA, enabling target enrichment and proving useful for studying gene-edited plants. For example, Huang and colleagues [35] conducted research using PacBio HiFi sequencing to analyze MON810 samples, implementing methods that enhanced sequencing precision and revealed complete sequences. This technique allowed for precise identification and quantification of genetic alterations at 0.1%.

An overview of biological systems based on the impact of gene editing on traits linked to phenological characteristics, metabolomics, and proteomics can offer further insights into genetic modifications using NGTs. For instance, Atlas by omics comprehension can provide biomarkers for disease detection [36]. Looking ahead, advanced biosensing innovations based on chip-based techniques, such as SNP chips, promise high-throughput sensitivity, repeatability, and automation capabilities for NGT detection. These developments are expected to enhance specificity, robustness, and cost-effectiveness for identifying and studying genetic alterations. Furthermore, collaboration focused on regulatory frameworks and publicly accessible knowledge regarding target and off-target sequences is vital for accurate detection and regulation of genetic modifications. The alliance between developers and regulators represents a proactive approach that aligns with the need for robust regulatory frameworks, safety, and traceability in ensuring effective market oversight.

### 3.3. State of the Art for NGT Detection Methods in the Context of the European Regulations

Recent advances in detection methods have prompted discussions regarding the need to reassess existing techniques and explore alternatives that align with European regulatory guidelines. This presents a significant challenge in the rapidly evolving field of genetic modification. The European Network of GMO Laboratories (ENGL) establishes the standards for validating these detection methods. Once accredited, laboratories utilize these methods along with Certified Reference Materials (CRMs) for calibration and quality control [2,37,38].

The regulatory framework for introducing GMOs, including in food and feed products, mandates comprehensive risk assessments and the development of event-specific detection methods. This process involves conducting inter-laboratory studies to verify the consistency of detection methods across various accredited laboratories [39].

However, advancements in plant genetic breeding are impacting both scientific and regulatory frameworks. The limitations of traditional qPCR for the specific detection of single nucleotide polymorphisms (SNPs) necessitate a fundamental re-evaluation of procedures and an exploration of approaches that meet specificity requirements. Under European regulations, ENGL has highlighted the need to distinguish between natural and artificial variations [40,41] and has focused on developing methods to detect, identify, and quantify organisms [42,43].

Our investigation demonstrates that the *grf1-3* LNA method effectively detects and quantifies gene-edited Arabidopsis. To achieve complete specificity, this method should be complemented by another analytical approach and be evaluated on a case-by-case basis. Current European agreements take a precautionary approach aligned with European Union Directive 2001/18/EC, consistent with the Convention on Biodiversity and biosafety for food, feed, the environment, and other target organisms.

In addition, our study emphasizes the need for continued method development and rigorous validation, not only in the context of European regulation, but also for food safety and analytical methods for foodstuffs, which require robust and specific detection methods to distinguish between genetically modified and naturally occurring DNA variants. This is crucial for ensuring compliance with food safety, labeling, and traceability requirements.

The study’s results have significant implications for the regulation of genome-edited food crops, and the demonstrated difficulties in achieving complete specificity with qPCR methods underscore the need for comprehensive detection strategies that go beyond single-plex assays. The development and validation of robust, sensitive, and specific detection methods are critical for ensuring public trust and safety in gene-edited foods. The findings highlight the importance of a multi-targeted approach, possibly incorporating multiplexing, next-generation sequencing, and other analytical methods to ensure the accuracy and reliability of GMO detection. This will be especially important as more gene-edited crops reach the market.

Looking ahead, the development of improved and validated detection methods, for example, ddPCR and NGS, is crucial for effective regulatory oversight and risk assessment. These advanced techniques may provide higher sensitivity, specificity, and accuracy for detecting gene edits, particularly at low concentrations or in food matrices. Furthermore, international collaborations between regulatory agencies, researchers, and industry stakeholders are essential for harmonizing detection methods, developing consensus standards, and ensuring effective monitoring. This will ultimately contribute to consumer safety and informed choices regarding gene-edited food products.

With the potential for breeding programs utilizing NGT techniques, ensuring accurate detection, identification, and event specificity becomes crucial for regulatory compliance. Recognizing the strengths and limitations of current methods underscores the importance of (1) ensuring access to reliable genomic databases; (2) obtaining precise genomic single nucleotide variant (SNV) sequences for closely related species within the same genus, cultivars, or varieties; (3) providing comprehensive open genetic information from proponents of NGT varieties to support tracking of food and feed; and (4) identifying mutation signatures, such as insertions/deletions or SNVs, as well as natural selection pressures at specific loci for each new gene-edited plant event.

## 4. Material and Methods

### 4.1. Arabidopsis Germplasm

The *Arabidopsis thaliana* ecotype Columbia (Col-0) genotype and the mutant genotype for the Growth Regulatory Factor 1 (GRF1; Gene ID 826479) gene were obtained from the Eurasian Arabidopsis Stock Center (NASC; https://arabidopsis.info/CollectionInfo?id=49; accessed on 23 March 2025). The mutant, named *grf1-3* (NASC accession number N72426), is genetically identical to Col-0 (NASC accession number N1092) and was created by modifying the GRF1 gene using the CRISPR-Cas9 floral immersion protocol [44]. The *grf1-3* has a guanine (G) insertion at position 9,729,886 on the positive strand of exon 2 within the mRNA sequence encoding the GRF1 gene. At the chromosomal level, this mutation is located at Locus AT2G22840 on chromosome 2. Sanger sequencing for this locus was performed to confirm the mutation. The results of the sequencing analysis are available in a previous study [6].

### 4.2. Extraction of Genomic DNA

Seeds of Arabidopsis genotypes (Columbia; grf-1, 3, 4, and 8) were sown into pots containing sterile soil imbibed in water. Pots were kept at 4 °C under dark to break dormancy. After a week, pots were transferred to a growth chamber (Enviro Plant^®^) with a constant temperature regime of 25 °C, a 10 h photoperiod (160 µmol m^−2^ s^−1^), and 60% relative humidity. Leaves from 30-day-old Arabidopsis seedlings were harvested and frozen immediately with liquid nitrogen and stored at −70 °C for further use. Arabidopsis leaves (0.3 mg) were ground into powder using liquid nitrogen and used for genomic DNA extraction. Briefly, 400 µL of a Plant DNAzol^TM^ (Invitrogen^TM^, Santa Clara, CA, USA) reagent and 10 µL of RNase were added to ground samples. Samples were vortexed briefly and kept at 65 °C in a dry bath for 25 min, followed by centrifugation at 14,000 rpm for 5 min at room temperature. After centrifugation, the supernatant was collected in a fresh tube, and an equal volume of phenol/chloroform/isoamyl alcohol (25:24:1) (Invitrogen^TM^, Santa Clara, CA, USA) was added and mixed well by inverting the tubes. Following the centrifugation, the resulting upper aqueous layer was mixed with an equal proportion of pre-chilled isopropanol and incubated at −20° C for 3–12 h to precipitate the DNA. After incubation, the tubes were centrifuged at 14,000 rpm for 7 min, and DNA pellets were collected. The isolated DNA pellets were washed twice with 0.7 mL of pre-chilled 70% ethanol, followed by a spin at 14,000 rpm for 5 min at 4 °C. Then, the DNA pellets were air-dried at RT for 4 h or overnight and resuspended with 50 µL of nuclease-free water or a TE buffer. The DNA yield was measured using NanoDrop^TM^ 2000c (Thermo Scientific^TM^) and Qubit^®^ dsDNA HS (High Sensitivity) (Thermo Scientific^TM^). The quality was checked by resolving the DNA on 0.8% agarose gel electrophoresis.

### 4.3. Oligonucleotides Primers and Probes

Oligonucleotides were designed considering penalties such as GC content and secondary structures as well as other parameters for optimal activity (i.e., primers’ annealing temperature ranged from 56 °C to 64 °C; probe from 62 °C to 68 °C) using the Primer3Plus software (version: 3.3.0; https://www.primer3plus.com/index.html; accessed on 23 March 2025) from [45]. A locked nucleic acid (LNA)-containing primer system (herein named the LNA system) was tested for its specificity at the mutation site and compared to a generic TaqMan^®^ system (herein named the Taqman system). LNA modification was introduced in the 3′ end of the forward primer (herein named +G modification). The primer and probe set was designed to amplify a control for endogenous protein oxoglutarate (2OG) and Fe (II)-dependent oxygenase superfamily protein (herein named taxon system) designed by [46] and amplify a 209 bp fragment (Gene ID: AT1G03400 2) of *A. thaliana*. DNA primers and TaqMan™ probes were synthesized by Applied Biosystems™ (Life Technologies, Pleasanton, CA, USA), except for the locked nucleic acid (LNA)-containing primer, which was obtained from QIAGEN Genomic Services™ (Hilden, Germany). The probes were labeled with 6-carboxyfluorescein (FAM) at the 5′ end and non-fluorescent quencher (QSY or BHQ1) at the 3′ end.

### 4.4. Real-Time Quantitative PCR Method Development

A DNA serial dilution was established based on a Arabidopsis haploid genome size of 135 Mbp [47] and a DNA molecular weight mass (660 Da/bp) corresponding to 2.64 pg. A 5 µL DNA template was used in the PCR, with concentrations ranging from 10^5^ to 10^2^ copies (0.2 copies) diluted in IDTE (10 mM Tris, 0.1 mM EDTA; Integrated DNA Technologies™). The Kapa Probe Fast qPCR Master Mix (2×) Universal was used for all three systems (Taqman, LNA, and Taxon).

Two different cycling conditions were tested to determine optimal Cq adherence: the manufacturer’s conditions (initial denaturation step at 95 °C for 10 min, followed by 45 cycles of denaturation at 95 °C for 2 s and annealing/extension at 60 °C for 20 s) and an ENGL condition based on the latest validation protocols (initial denaturation step at 95 °C for 10 min, followed by 45 cycles of denaturation at 95 °C for 15 s and annealing/extension at 60 °C for 60 s). Both cycling protocols were tested using a reaction volume of 25 µL, containing forward and reverse primers at different concentrations. A fixed concentration of 20,000 DNA copies [588 pg/µL] of the corresponding target was used in the different testing conditions.

For the LNA system, the specific single-point insertion was used as a strategy to place the LNA modification and, therefore, differentiate the amplification from the WT genotype. Primer and probe titration tests were performed to establish the optimal Ct adherence. For the oligonucleotide’s primers, we used 200 nM, 400 nM, and 600 nM in all possible combinations for forward and reverse in the first titration assay. The probe was titrated in 100 nM, 200 nM, and 300 nM. A second titration assay was conducted using 800 nM, 1000 nM, and 1200 nM for the oligonucleotide’s primers and 400 nM, 500 nM, and 600 nM for the probe. Temperature gradients tested ranged from 56 °C up to 66 °C.

The taxon system was modified from [46] to allow running samples in the same PCR plate. Optimized conditions were the initial denaturation step at 95 °C for 10 min followed by 45× 15 s at 95 °C and 60 s at 62 °C with primer concentrations of 400 nM forward, 600 nM reverse, and 200 nM probe (Table 9).

### 4.5. Inter-Laboratory Method Validation

Two laboratories participated in the inter-laboratory validation: the Laboratory of Developmental Physiology and Plant Genetics at the Federal University of Santa Catarina in Brazil (UFSC) (herein named Laboratory 1) and the GMO Laboratory at the Environmental Agency Austria (EEA) (herein named Laboratory 2). The initial testing requirements followed the JRC Minimal Performance Criteria [18]. Standard solutions were tested in triplicates for both the *grf1-3* system and the taxon system. No template control (NTC) consisting of nuclease-free water was also applied in triplicate.

#### 4.5.1. Trueness and Precision Parameters

According to Figure 2 of the ENGL guideline on method verification, trueness and precision parameters were evaluated [16]. For qPCR reactions, a multifactorial experimental design was implemented across four plates. Each plate contained two replicates of *grf1-3* DNA concentrations (10%, 1%, and 0.1%). Additionally, precision was calculated using a calibration curve comprising five points (Supplementary Material S2). The mean Ct values and standard deviations were utilized to calculate the relative standard deviation (RSDr).

#### 4.5.2. Robustness Parameters

Robustness was determined using a multifactorial robustness assay according to [17] in eight different combinations. Robustness testing was examined by running the PCR systems in ABI 7500 and BioRad CFX96 qPCR machines. In addition, the KAPA PROBE FORCE qPCR Master Mix and QuantiTect Multiplex qPCR Kit were tested. Annealing temperatures were modified by ±1 °C, master mix volumes by ±5%, and primer/probe concentrations were reduced by 30%.

#### 4.5.3. LOD-LOQ Parameters

LOD and LOQ were determined according to [16,17]. The LOD-LOQ assay for the *grf1-3* PCR system was conducted with the preparation of Standard 1 of a 1:10 dilution of *grf1-3* DNA (~40 ng/µL measured in Qubit, DNA broad spectra ThermoFischer) in salmon sperm DNA background (20 ng/µL) following [48]. Five dilution points (2500, 500, 100, 20, 10) were used in triplicate, and six extra dilution points (40, 20, 10, 5, 1, 0.1) were used in 12 replicates. The LOD was determined as the lowest amount of analyte with all replicates positive, and LOQ was determined based on the standard deviation of 12 replicates ≤25%. The comparison of Ct mean values, standard deviation values, repeatability deviation values (RSDr), amplification efficiency (E), and R^2^ was performed.

#### 4.5.4. Specificity Testing

The in silico studies were conducted using the JRC GMO-Matrix database [18,49]. This involved matching primers and probes with GMO sequences. However, NGT products pose an extra challenge to this limited analysis as endogenous plant genes are modified without transgenic insertions. Therefore, a comprehensive genome database survey was performed. Primer-BLAST and BLASTN (NCBI) analyses were performed to assess sequence similarity against the amplicon sequences of *grf1-3* (AT2G22840; Chr 2). This in silico analysis was previously performed by [6].

In vitro specificity was assessed on DNA of various non-GM plants or Certified Reference Materials (CRMs). The following genotypes were tested: oilseed rape (GT73, MS8, RF3, 88302, 73496, and non-GM canola) and Arabidopsis (*grf1-3*, col WT, grf8-61, grf4-6, grf3-17). For reproducibility, both *grf1-3* and taxon PCR systems were tested in the same plate with background DNA (100 ng/PCR of salmon sperm DNA). At least 2500 copies of the test DNA were added to each PCR reaction.

## 5. Conclusions

In conclusion, the rapid advancement of detection methods for genetically modified organisms (GMOs) necessitates a thorough reassessment of existing techniques to meet the evolving European regulatory standards. The pressing need to differentiate between small DNA variations underscores the importance of developing gene-editing event-specific detection methods.

The technical challenges associated with the *grf1-3* LNA method for detecting and quantifying gene-edited Arabidopsis have been highlighted. Our case study demonstrated that the *grf1-3* method is capable of amplifying Brassica DNA templates, with Cq values around 35. However, the slope was lower compared to native NGT, and the delta Rn was approximately 1. Therefore, while the method is not specific, it is recommended that taxon-specific primers be used to differentiate Arabidopsis from other genuses.

To distinguish between native mutants and SNP mutants resulting from NGT techniques, the use of sequencing tools or databases containing key sequences of the edited genotype might be needed. Identifying similar genomic sequences homologous to the GM event is crucial for the specificity prediction when developing a detection and quantification strategy for NGTs.

Given the lack of international consensus on regulations for GMOs obtained by NGTs, it is vital to adopt combined methodologies. These may include techniques such as digital PCR methods, sequencing approaches, and/or other genomic profiling analysis. Such strategies will be essential for addressing the complexities of detecting gene-edited organisms and ensuring compliance with regulations.

The relevance of new harmonized analytical methods will also ensure continuous contribution to consumer safety and informed choices regarding gene-edited food products. Furthermore, these methods will contribute to the demand for DNA analysis for food safety, such as the detection of food containing harmful bacteria, viruses, parasites, or any other infectious, toxic, or allergenic substances that might be present in foodstuffs.

Continuous collaboration among regulatory agencies, academic institutions, and industry stakeholders is vital for refining detection strategies and expanding the methodologies available for NGT labeling. Additionally, establishing reliable genomic databases, obtaining precise single nucleotide variant sequences, and ensuring comprehensive genetic information sharing will support effective monitoring and tracking of NGT products. Given the potential implications of deploying NGT organisms, especially in agriculture, stakeholders must remain vigilant about the safety and environmental impact of these technologies. Ultimately, advancing detection techniques and regulatory frameworks will foster public trust and ensure the safe integration of gene-edited organisms into the market.

## Figures and Tables

**Table 1:** Summary of the LNA oligonucleotide primer first titration experiment.

Experiment	Forward Primer Concentration (nM)	Reverse Primer Concentration (nM)	Probe Concentration (nM)	Annealing Temperature (°C)	Cq Value	Fluorescence (ΔRn)
1	200	200	200	60	26.02	~2
2	200	400	200	60	24.35	~2
3	200	600	200	60	24.49	2
4	400	200	200	60	23.14	~2
5	400	400	200	60	22.03	~3
6	400	600	200	60	22.19	~3
7	600	200	200	60	22.53	2
8	600	400	200	60	21.46	4
9	600	600	200	60	21.49	5

**Table 2:** Summary of the LNA oligonucleotide primer second titration experiment.

Experiment	Forward Primer Concentration (nM)	Reverse Primer Concentration (nM)	Probe Concentration (nM)	Temperature (°C)	Cq Value	Fluorescence (ΔRn)
1	800	800	400	60	23.60	~6
2	800	1000	400	60	23.04	~10
3	800	1200	400	60	23.93	~6
4	1000	800	400	60	23.55	~7.5
5	1000	1000	400	60	22.97	~11
6	1000	1200	400	60	23.36	~9
7	1200	800	400	60	23.37	~8.5
8	1200	1000	400	60	23.46	~9
9	1200	1200	400	60	23.04	~12 (replicates variation)

**Table 3:** Results for the limit of detection (LOD) parameter for the *grf1-3* edited line in the established PCR quantitative method based on LNA primer modification.

LOD		
Nominal Copy Number of Target Sequence	N° of Replicates	N° of Positive
40	12	12
20	12	12
10	12	12
5	12	12
1	12	9
0.1	12	2

**Table 4:** Results for the multi-experimental design for robustness parameter validation.

Factor-Level Combination								
Condition	1	2	3	4	5	6	7	8
Real-time PCR equipment	ABI7500	ABI7500	ABI7500	ABI7500	CFX96	CFX96	CFX96	CFX96
PCR reagent kit	KAPA	KAPA	Qiagen	Qiagen	KAPA	KAPA	Qiagen	Qiagen
Annealing temperature (°C)	63	61	63	61	63	61	63	61
Master mix volume (µL)	19	19	21	21	21	21	19	21
Primer concentration (nM)	1200	840	1200	840	700	1000	700	1000
Probe concentration (nM)	400	280	280	400	280	400	400	280

**Table 5:** Trueness and precision measurements for different DNA concentration samples.

Target % *grf1-3* Level	Measured % *grf1-3* Level	Precision (RSDr%)	Bias % of Target *grf1-3* Level
0.1	0.09	15.10	−8.23
1	1.106	7.58	10.60
10	10.154	6.16	1.54

**Table 6:** Performance of the *grf1-3* method for the LOQ assay.

LOQ			
Nominal Copy Number of Target Sequence	Copy Number Measured	SD	RSDr
40	48.4	9.9	20.6
20	24.5	9.3	37.9
10	14.1	5.2	36.5
5	6.9	3.6	52.5
1	2.2	1.3	58.3
0.1	1.2	0.2	16.5

**Table 7:** Sequence similarity between genomic sequences and the *grf1-3* oligonucleotide primer sequences as investigated in Zanatta et al. [5].

Species Hits	Gene ID	Forward Oligonucleotide Sequence	Reverse Oligonucleotide Sequence	Probe Sequence
*Brassica napus*	XM_0487528; XM_0138297; HG994367.1; HG994357.1.	GGAAAGAAATGGCGGTGCT-	GCCACAATACTAATGCTaCCG	ACAGAGGCCGCCATCGTTCA
*Brassica rapa*	LS974619.2	GGAAAGAAATGGCGGTGCT-	GCCACAATACTAATGCTaCCG	ACAGAGGCCGCCATCGTTCA

Note: In the LNA *grf1-3* method, the forward primer includes +G, with the sequence 5′-GGAAAGAAATGGCGGTGCT+G-3′. The reverse primer sequence is 3′-CGGTGTTATGATTACGACGGC-5′.

**Table 8:** Mean Cq values for the *grf1-3* and the taxon-specific PCR system.

Sample	DNA [] (ng)	Target Gene	Average Cq	Target Gene	Average Cq
grf3-9	15	*grf1-3*	36.7	RG	21.8
grf4-17	15	*grf1-3*	34.7	RG	21.7
grf8-61	15	*grf1-3*	41.3	RG	26.79
*grf1-3*	15	*grf1-3*	18.9	RG	21.2
Col-01	15	*grf1-3*	35.8	RG	21.9
GT73	100	*grf1-3*	42.88	cruA	26.2
MS8	100	*grf1-3*	37.16	cruA	21.55
RF3	100	*grf1-3*	37	cruA	25.47
88302	100	*grf1-3*	43.21	cruA	25.49
73496	100	*grf1-3*	37.75	cruA	22.61
non-GM oilseed rape	100	*grf1-3*	40.72	cruA	25.11

**Table 9:** Quantitative PCR methods used in this study. The table includes the sequences of primers and oligonucleotide probes, the positions of locked nucleic acid (LNA) modifications, PCR conditions, and corresponding bibliographic references. The fluorophore used was 6-carboxyfluorescein (FAM), and the quencher was a succinimidyl ester-based quencher (QSY).

Primer Name	Primer and Probe Sequence	Amplicon Length	Primer and Probe Concentration	PCR Condition	Reference
*grf1-3* Taqman	F: 5′-GGAAAGAAATGGCGGTGCT-3′ R: 5′-CGGCAGCATTAGTATTGTGGC-3′ P: 5′-ACAGAGGCCGCCATCGTTCA-3′	130	600 nM forward 600 nM reverse 200 nM probe	95 °C 10 min; 95 °C by 15 s [45×]; 62 °C by 1 min	This study
*grf1-3* LNA	F: 5′-GGAAAGAAATGGCGGTGCT+G-3′ R: 5′-CGGCAGCATTAGTATTGTGGC-3′ P: 5′-ACAGAGGCCGCCATCGTTCA-3′	131	1000 nM forward 1200 nM reverse 400 nM probe	95 °C 10 min; 95 °C by 15 s [45×]; 62 °C by 1 min	This study
Taxon specific primer (RG (AT1G03400.1)	F: 5′-GCGGAGCATAGGGTGATAGC-3′ R: 5′-TGTAACTTAGGAGCATCGAGCG-3′ P: 5′-ATGGGCCAATCAAAGATCTCCTGTCTGC-3′	209	400 nM forward 600 nM reverse 200 nM probe	95 °C 10 min; 95 °C by 15 s [45×]; 62 °C by 1 min	[46]

## Data Availability

The data used to support the findings of this study can be made available by the corresponding author upon request.

## Supplementary Materials

The following supporting information can be downloaded at: https://www.mdpi.com/article/10.3390/ijms26073308/s1.
